# Effect of reused litter on early cecal microbiota succession and the immune cells distribution in layer chickens

**DOI:** 10.1371/journal.pone.0355745

**Published:** 2026-08-12

**Authors:** Nimra Khalid, Shuja Majeed, Bikas Raj Shah, Ali Nazmi

**Affiliations:** 1 Department of Animal Sciences, The Ohio State University, Wooster, Ohio, United States of America; 2 Food for Health Discovery Theme, The Ohio State University, Columbus, Ohio, United States of America; Ankara University: Ankara Universitesi, TÜRKIYE

## Abstract

This study was designed to evaluate early immune development and gut microbiome establishment in layer chicks reared on reused or fresh litter. A total of 160 newly hatched female Hy-Line W80 layer were housed on fresh or used litter (n = 80/group). Body weight was recorded weekly, ceca, ileum and spleen samples were collected at days 7 and 35 for gut microbiome analysis and intraepithelial lymphocytes (IELs) study. Results showed that layer chicks reared on fresh litter exhibited significantly higher body weight compared with layer chicks reared on used litter from day 14 to day 35 (P < 0.05), while mortality remained low and was not significantly different between groups. Cecal microbiota analysis revealed significant litter-associated differences in alpha and beta diversity at day 7 (P < 0.05). However, by day 35, microbial richness and overall community composition were no longer significantly different between treatments. At the phylum level, Bacillota was the dominant phylum in both litter groups at days 7 and 35. Pseudomonadota was the second most abundant phylum at day 7, whereas Bacteroidota became the second most abundant phylum by day 35. This shift indicates age related maturation of the cecal microbial community. In terms of immune responses, no significant difference was found in IEL subsets at day 7 between litter groups. However, by 35 days, the used litter exposure resulted in a significant expansion of TCR ⁻ , TCRγδ ^+^ , TCRαβ, TCRαβ ⁺ CD8αβ⁺ and TCRαβ ^+^ CD8αα ^+^ IEL subsets (P < 0.05). Similarly, splenic TCRγδ^+^ and TCRαβ ^+^ CD8αβ^+^ populations were significantly elevated in used litter layer chicks at day 35. These findings highlight that exposure to different litter environment was associated with distinct cecal microbial profiles and immune-cell distributions in layer chicks. Given the limited information available in laying hens, these results provide novel baseline data and support further studies investigating the role of litter-associated microbial exposure in shaping early immune development.

## Introduction

Litter is a principal component of poultry production systems, which serves simultaneously as bedding material, a microbial reservoir, and a source of continual environmental exposure for birds. It typically comprises bedding substrates, manure, feathers, spilled feed, and uric acid, forming a complex ecological niche in which environmental microorganisms, enteric bacteria, and potential pathogens coexist [1]. Litter-associated microbes play a major role in shaping early gastrointestinal colonization and immune development, as chickens are continuously exposed to litter through pecking, ingestion, and skin contact [2–5]. Fresh litter is generally dominated by environmental taxa such as *Lactobacillus*, whereas recycled litter becomes progressively enriched with intestinally derived microbial communities, including members of *Clostridiales* and butyrate-producing taxa such as *Faecalibacterium prausnitzii* [6–8].

Recycled litter is widely used in commercial broiler production, where litter may be reused for multiple successive flocks, often 5−15 cycles, before complete cleanout [2,9,10]. This production model allows litter-associated effects on growth, microbiota composition, and immune responses to be evaluated within a relatively short and economically feasible experimental timeframe. Previous broiler studies have demonstrated that litter reuse can significantly influence both intestinal microbiota composition and immune responses [2,11]. The microbiota shifts have been associated with altered immune phenotypes, including increased serum nitric oxide levels, enhanced antibody responses, changes in splenic lymphocyte proliferation, and modifications in intestinal and systemic lymphocyte subpopulations [2]. Experimental studies further indicate that recycled litter can skew intestinal immune responses toward a more inflammatory profile, with increased expression of cytokines such as IL-1 and IL-4 and altered regulatory T-cell (Treg) populations in the cecal tonsils [7]. Collectively, these findings support the concept that litter-associated microbial exposure plays a key role in shaping post-hatch immune development in broilers.

In contrast, information regarding the effects of litter reuse on gut microbiota and immune-cell populations in layer chickens remains largely unavailable. Layer chickens have substantially longer lifespans (72–80 weeks) and are housed in more variable housing systems, including cage, cage-free, and aviary environments, where litter exposure may persist throughout extended rearing and laying periods [12,13].

These logistical challenges have limited controlled experimental studies on litter reuse in layers. In addition, As the poultry industry continues to transition toward cage-free production systems, understanding how litter management practices influence the gut microbiome and immune system during early life has become increasingly important. However, controlled studies evaluating the impact of fresh versus reused litter on both cecal microbiota composition and immune-cell populations in layer chickens are scarce. Therefore, the present study was designed to evaluate the effects of fresh and reused litter on cecal microbial communities and immune-cell distribution in layer chicks. We hypothesized that exposure to reused litter would result in distinct cecal microbial communities and immune-cell distributions compared with fresh litter.

## Materials and methods

### Ethics statement

All animal experiments were carried out in the Wooster Poultry Research Center in accordance with The Ohio State University Institutional Animal Care and Use Committee of the Ohio State University (2024A00000066). Chicks were monitored daily for general health, behavior, access to feed and water, signs of distress, morbidity, and mortality. Litter condition and room environment were routinely observed as part of animal welfare monitoring. Any birds showing severe distress or illness were to be removed and humanely managed according to the approved IACUC protocol.

### Litter treatment

The used litter was obtained from a previous cage-free layer flock that was reared in the OSU Poultry Research Center in Wooster. The litter was heaped in long rows at the center of the pen to initiate a heat cycle for five days [14,15]. Crusted and compacted material was broken to enhance aeration and moisture regulation. Following this process, the litter was evenly distributed across the pen floor. A separate floor pen was covered with fresh litter (wood shavings).

### Animals and experimental design

A total of 160 newly hatched female Hy-Line W80 layer chicks were obtained and raised according to the management guidelines (Hy-Line North America, West Des Moines, IA, USA). Chicks were randomly housed in environmental controlled floor pens containing either used litter (n = 80) or a fresh litter (n = 80) at stock density of 0.046 m^2^/chicks. Throughout the study, all layer chicks were fed a standard non-medicated poultry diet formulated by The Ohio State University feed mill. The ingredient composition and formulated nutrient analysis of the diet are presented in Table 1. Feed and water were provided ad libitum throughout the study. Chicks were weighed at day 1 of age and weekly. On days 7 and 35, a subset of layer chicks (n = 8 per group) was euthanized using CO_2_ asphyxiation for sample collections. Ceca (n = 5 per group) were removed, and their contents were collected in sterile Whirl-Pak bags and immediately stored at –80 °C until processing. In addition, ileums and spleens (n = 8/ per group) were harvested for immune cell isolation and flow cytometry analysis. Mortality was monitored throughout the study.

**Table 1:** Ingredient composition and calculated nutrient content of the experimental diet fed to layer chicks.

Ingredient	Percentage (%) in feed
Corn	58.74
Soybean meal	32.1
Meat and bone meal, 50%	2.5
Animal fat	3.08
Limestone, fine	1.2
Dicalcium phosphate, 18.5% P	1.23
Salt	0.22
Sodium bicarbonate	0.18
DL-Methionine, 99%	0.3
L-Lysine, 99%	0.13
L-Threonine	0.07
Choline chloride, 60%	0.1
Vitamin-mineral premix without choline	0.15
Total	100
**Calculated nutrient composition**	
**Nutrient**	**Calculated Value**
Metabolizable energy	3000 kcal/kg
Crude protein	20.01%
Total lysine	1.20%
Total methionine	0.62%
Total methionine + cystine	0.94%
Total threonine	0.84%
Calcium	1.05%
Available phosphorus	0.45%
Sodium	0.18%
Chloride	0.18%
Choline	2000 mg/kg
Crude fat	5.45%
Crude fiber	3.37%

### DNA extraction, 16S rRNA sequencing and microbiome analysis

Genomic DNA was extracted from 5 cecal contents per group, using the Mag-Bind® Blood & Tissue DNA HDQ 96 Kit (Omega Bio-Tek, USA) according to the manufacturer’s instructions. DNA concentration and purity were verified using a NanoDrop spectrophotometer. Cecal microbial DNA was further processed for bacterial community profiling by sequencing the V3-V4 region of the 16S rRNA gene using an Illumina platform with paired-end reads. Raw sequencing reads were processed using the QIIME pipeline (v1.9.1). Sequences were quality-filtered, merged, and screened for chimeras prior to clustering into operational taxonomic units (OTUs) at 97% sequence similarity using a de novo clustering approach. Taxonomic assignment was performed against the SILVA database (v138). Alpha diversity indices, including Observed OTUs and Shannon index, were calculated to assess within-sample diversity. Beta diversity was evaluated using Bray-Curtis dissimilarity, and differences in microbial community structure were visualized using principal coordinates analysis (PCoA). Statistical significance among groups was assessed using PERMANOVA, with P < 0.05 considered statistically significant. Differentially abundant taxa between litter treatments were identified using linear discriminant analysis effect size (LEfSe). Taxa with an LDA score greater than 2.0 and a P-value < 0.05 were considered significantly enriched.

### Immune cell isolation and flow cytometry

At 7 and 35 days of age, Intraepithelial lymphocytes (IELs) were isolated from ileal tissue (n = 8 per group) using a previously established protocol [16]. Briefly, approximately 1-cm fragments of ileal tissue were subjected to mechanical dissociation by agitation at 150 rpm for 45 minutes at 37 °C in phosphate-buffered saline (PBS) supplemented with 5% chicken serum, 2 mM dithiothreitol, and 2 mM EDTA. The resulting cell suspension was enriched using a 40/70% Percoll density gradient (Cytiva, Marlborough, MA, USA). The spleen was gently crushed and passed through a 45-µm cell strainer (Corning, NY) to obtain a single-cell suspension. Splenocytes were then enriched by density gradient centrifugation over Histopaque (1.077 g/mL; Sigma-Aldrich, St. Louis, MO) at 1,200 × g for 10 minutes at 10 °C without brake. Recovered cells from ileal and splenic tissues were washed, resuspended in staining buffer, and counted using the trypan blue exclusion method. Finally, isolated cells were stained with fluorochrome-conjugated anti-chicken CD45 SPRD (LT40), CD4 PE-CY7 (CT-4), CD3 AF547 (CT-3), TCRγδ FITC (TCR-1), CD8α AF700 (CT-8), and CD8β PE (EP42) antibodies (SouthernBiotech, Brimingham, Al, USA), and Ghost viability dye-Red 510 (Tonbo Biosciences, San Diego, CA, USA) Cells were acquired on NorthernLights^TM^ flow cytometry (Cytek, Fremont, CA, USA). Fluorescence compensation was performed using single-stained controls for each fluorochrome, with unstained cells included to assess cellular autofluorescence. Immune cell populations were identified and gated according to a previously established strategy [16]. Data acquisition and analysis were conducted using FlowJo™ software (version 10.10.0; BD Biosciences, NJ, USA).

### Statistical analysis

All statistical analyses were performed using GraphPad Prism v10.0.03 (GraphPad Software, Boston, MA, USA). Litter treatments were applied at the room/pen level, and individual layer chicks sampled within each treatment were used for outcome measurements. Data was assessed for normality using the Shapiro-Wilk test. Normally distributed body weight data were analyzed using an unpaired Student’s t-test according to the model: Yᵢ = μ + Tᵢ + εᵢ, where Yᵢ is the observed response, μ is the overall mean, Tᵢ is the effect of litter treatment, and εᵢ is the residual error. Flow cytometry data that did not meet normality assumptions were analyzed using the Mann-Whitney U test. Statistical significance was set at *p < 0.05*.

## Results

### Growth performance

**Fig 1:**
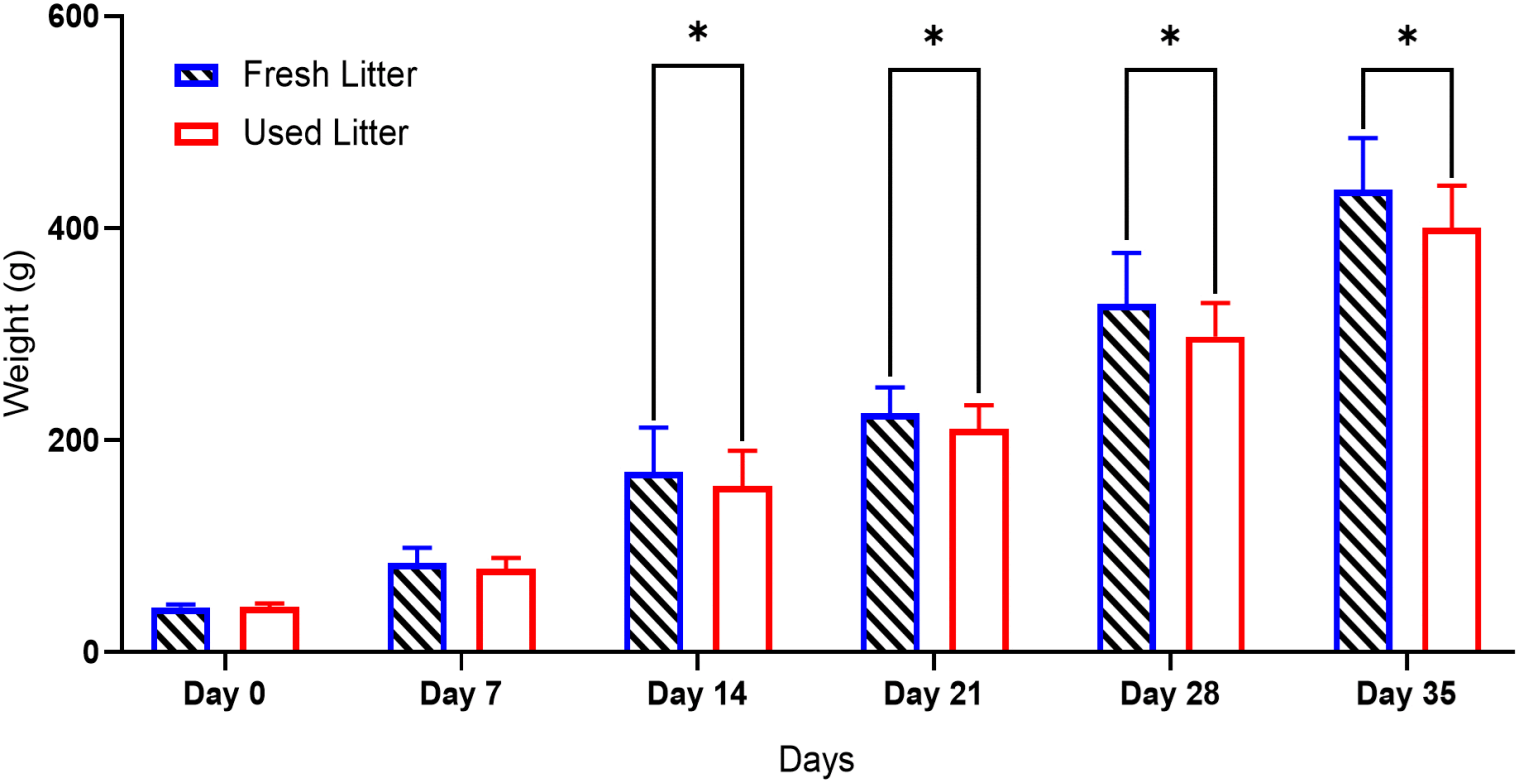
Effect of fresh vs used litter on body weight across the rearing period in layer chicken. Data are presented as mean ± SD. Statistical significance was determined using the t test. * p < 0.05.

Layer chicks reared on fresh litter showed higher body weight than with those reared on used litter (Fig 1), with significant differences observed on day 14 and persisting through day 35 of age (P < 0.05). Mortality during the first week of age was numerically higher in the fresh litter layer chicks (2.5%) on their first week of age, compared to the used litter layer chicks 1.25%; however, the difference was not statistically significant (p > 0.05). No additional mortality was recorded thereafter in either group.

### Cecal microbiota study

#### Sequencing output and quality metrics.

High throughput 16S rRNA gene sequencing generated sufficient depth and quality for downstream microbiome analyses. After quality filtering, approximately 2.7 million high-quality sequences were retained across all samples, with an average of 1.36 × 10⁵ sequences per sample. Read utilization rates exceeded 99.5%, indicating efficient sequence merging and minimal loss during quality control. Good’s coverage values were consistently high (>0.99), confirming that sequencing depth was adequate to capture the majority of bacterial diversity present in the samples.

### Alpha diversity

**Fig 2:**
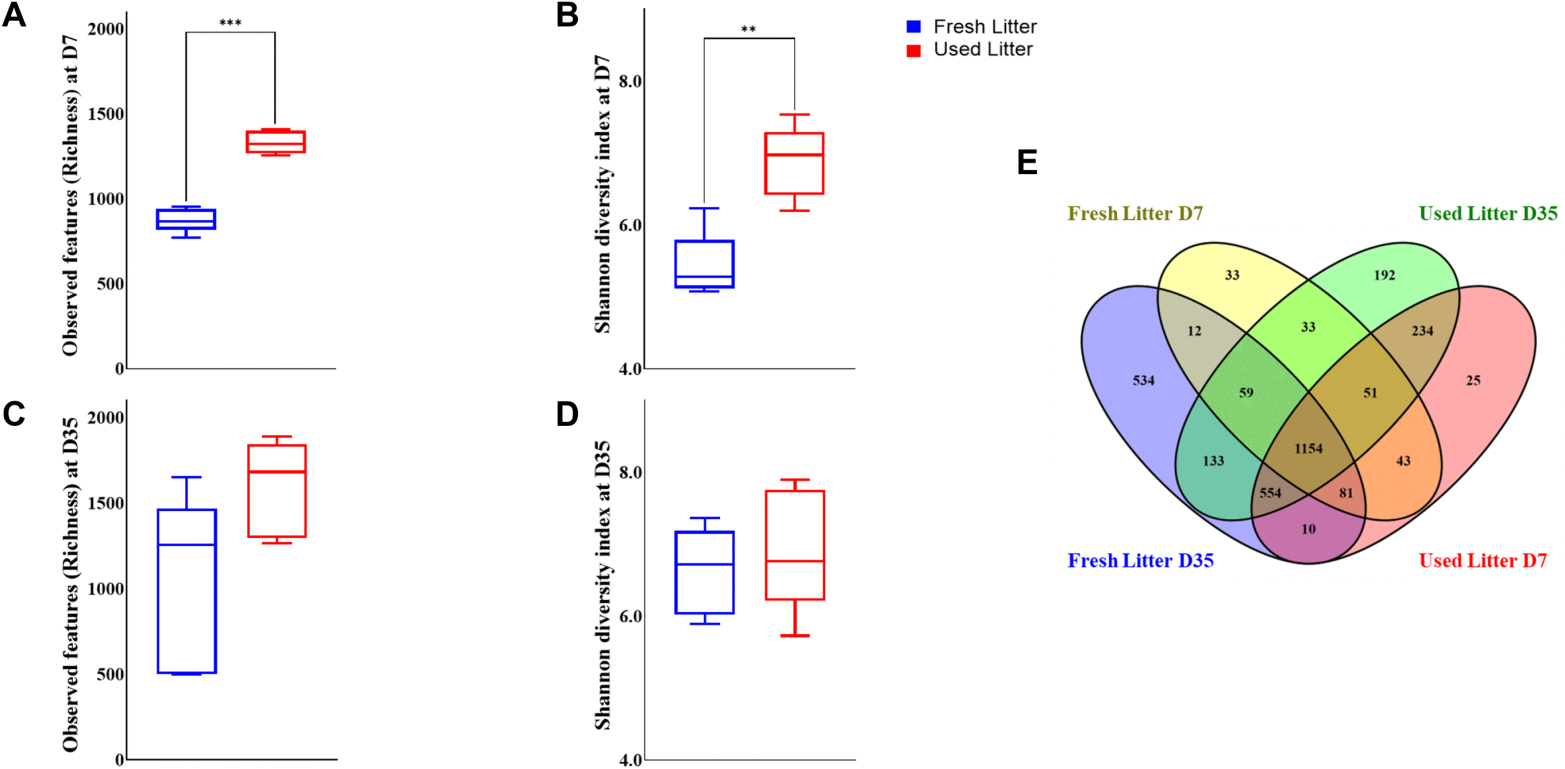
Alpha diversity and shared operational taxonomic units (OTUs) of the cecal microbiome in layer chicks reared on fresh and used litter at days 7 and 35. (A) Observed features at Day 7 between fresh vs used litter group. (B) Shannon index at Day 7 between fresh vs used litter group (C) Observed features at Day 35 between fresh vs used litter group. (D) Shannon index of Day 35 between fresh vs used litter group (E) Group specific OTUs. ** p < 0.01, *** p < 0.001.

Alpha diversity analysis revealed significant differences in microbial richness and diversity between layer chicks reared on fresh and used litter at day 7 (*P < 0.05*) (Fig 2A and B). However, by day 35, no significant differences were observed between litter types (Fig 2C and D), indicating convergence of microbial communities over time. Venn diagram analysis showed the distribution of shared and unique taxa among groups, identifying 1154 shared taxa across all groups, representing the core microbiome (Fig 2E). Fresh litter group at day 35 colonized with the highest number of unique taxonomic units (n = 534), followed by used litter at day 35 (n = 192), fresh litter at day 7 (n = 33), and used litter at day 7 (n = 25).

### Beta diversity

**Fig 3:**
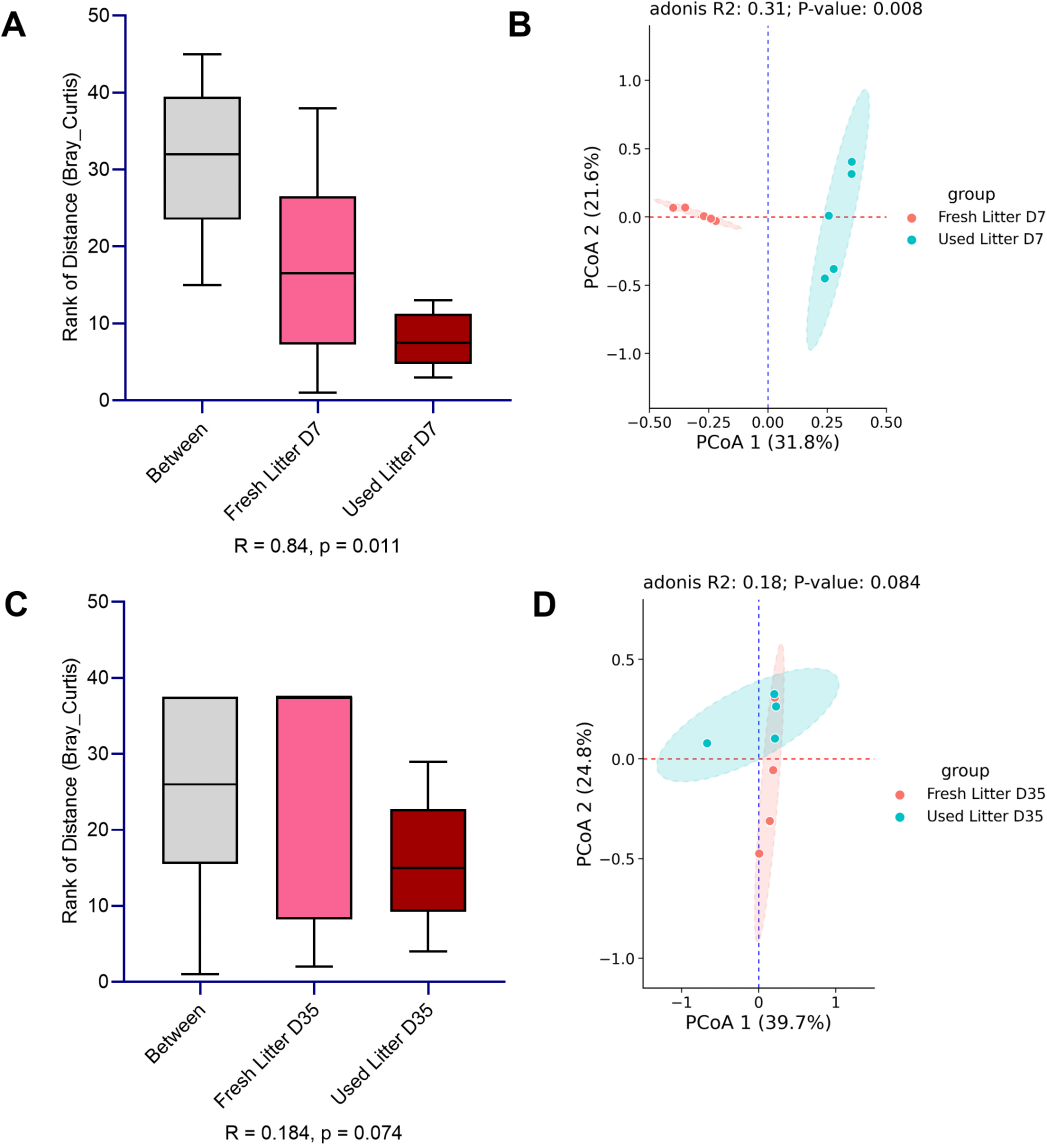
Beta diversity analysis of cecal microbial communities based on Bray–Curtis dissimilarity. (A) ANOSIM comparing between fresh litter and used litter at day 7 (B) Principal coordinates analysis (PCoA) of fresh vs used litter at day 7. (C) ANOSIM comparing between fresh litter and used litter at day 35. (D) PCoA of fresh vs used litter at day 35.

Beta diversity analysis revealed significant differences in microbial community composition between layer chicks reared on fresh and used litter at day 7, as demonstrated by analysis of similarities ANOSIM (R = 0.84, P = 0.011) (Fig 3A) and PERMANOVA (R² = 0.31, P = 0.008) (Fig 3B). In contrast, by day 35, microbial communities were no longer significantly different between the two litters (ANOSIM: R = 0.184, P = 0.074; PERMANOVA: R² = 0.18, P = 0.084) (Fig 3C and D), indicating convergence of community structure over time.

### Relative abundance

**Fig 4:**
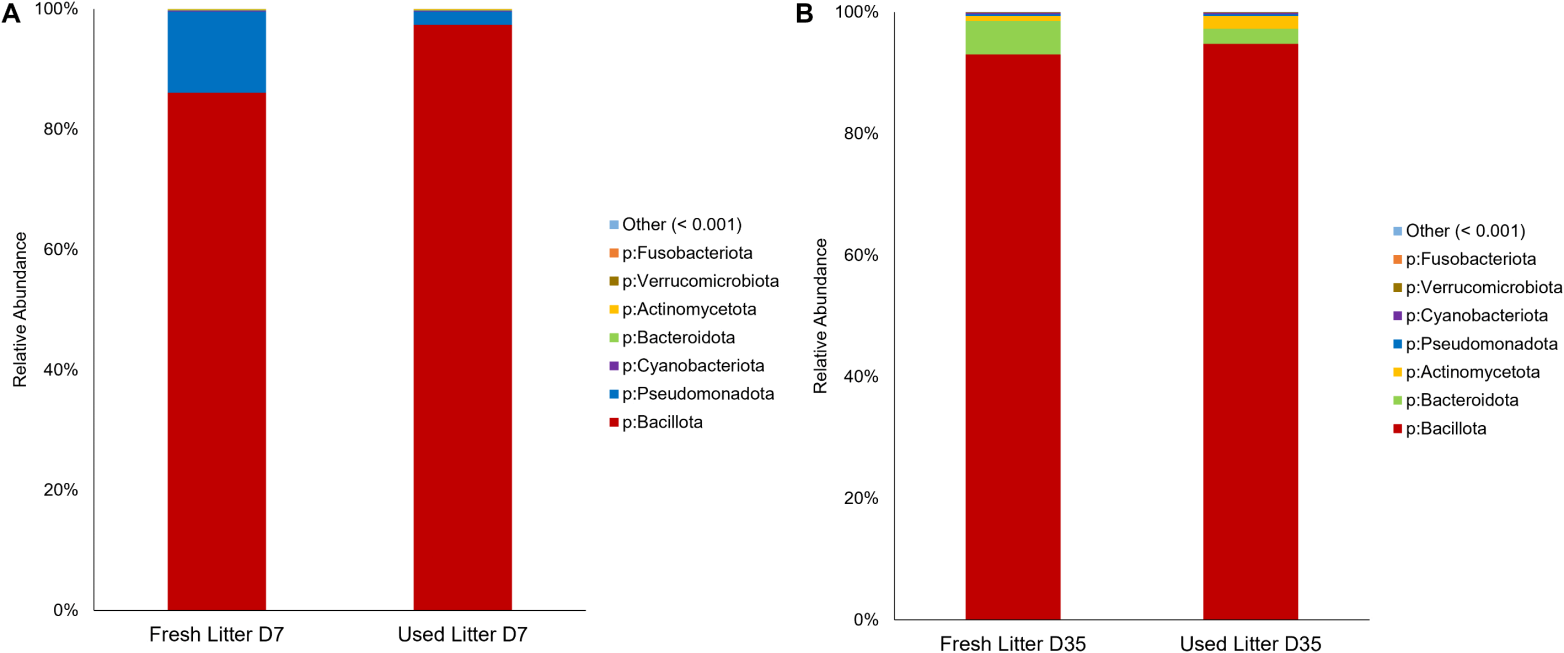
Relative abundance of dominant bacterial phyla in the cecal microbiota of layer chicks reared on fresh and used litter at day 7 and day 35. (A) Relative abundance of major bacterial phyla at day 7. (B) Relative abundance of major bacterial phyla at day 35. Low-abundance phyla (<0.001) were grouped as “Other (<0.001)”.

The relative abundance of dominant bacterial phyla in the cecal microbiota is shown in Fig 4. Across all treatments and sampling times, Bacillota was the predominant phylum. At day 7, the microbial communities of layer chicks reared on both litter types were primarily composed of Bacillota and Pseudomonadota. However, Cyanobacteriota were more abundant in layer chicks raised on fresh litter whereas Actinomycetota were more abundant in birds raised on used litter (Fig 4A).

By day 35, the microbial composition shifted, with Bacteroidota emerging as the second most abundant phylum in both litter treatments (Fig 4B). In layer chicks reared on fresh litter, the dominant phyla were Bacillota, Bacteroidota, Actinomycetota, and Pseudomonadota, whereas layer chicks raised on used litter showed a similar pattern but with Cyanobacteriota detected instead of Pseudomonadota among the dominant phyla.

### Differentially abundant taxa across litter type

**Fig 5:**
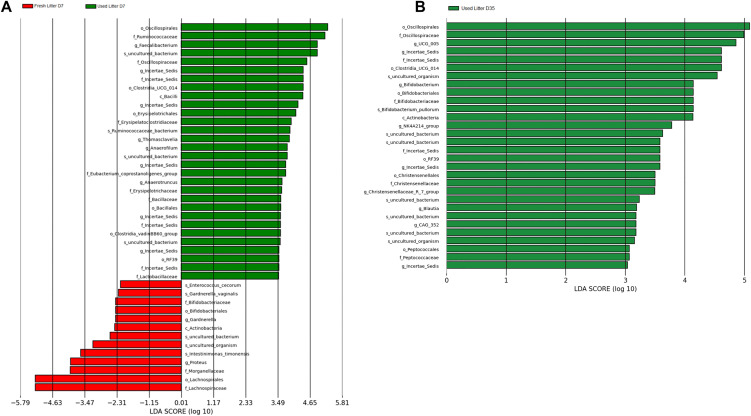
Differentially abundant bacterial taxa between litter types at day 7 and day 35. (A) Linear discriminant analysis effect size (LEfSe) identifying bacterial taxa differentially enriched between layer chicks reared on fresh litter and used litter at day 7. Taxa with a Linear Discriminant Analysis score (LDA) > 2.0 are shown. Positive LDA scores (green bars) indicate taxa enriched in the used litter group at day 7, whereas negative LDA scores (red bars) indicate taxa enriched in the fresh litter group at day 7. The magnitude of the LDA score reflects the effect size of each taxon in discriminating between litter treatments. (B) LEfSe identifying bacterial taxa differentially enriched between layer chicks reared on fresh litter and used litter at day 35. Taxa with a LDA score > 2.0 are shown. All displayed taxa (green bars) indicate enrichment in the used litter group at day 35, and no taxa in the fresh litter group met the LEfSe significance and LDA thresholds.

As both alpha and beta diversity analyses revealed significant differences between litter treatments at day 7, Linear discriminant analysis effect size (LEfSe) analysis was performed to identify the bacterial taxa contributing to these community differences. LEfSe (LDA > 2.0) revealed distinct taxa enriched in each litter treatment at day 7 (Fig 5A). layer chicks reared on used litter were characterized by enrichment of some notable taxa including Oscillospirales, Ruminococcaceae, and *Faecalibacterium*, whereas layer chicks reared on fresh litter showed enrichment of Lachnospiraceae, *Proteus*, *Enterococcus cecorum*.

At day 35, fewer taxa met the LEfSe significance threshold (Fig 5B). The taxa identified were enriched exclusively in the used litter group, with prominent representatives including Oscillospirales, Oscillospiraceae, and *Bifidobacterium*, while no taxa were significantly enriched in layer chicks reared on fresh litter at this time point.

### Immune cell dynamics

**Fig 6:**
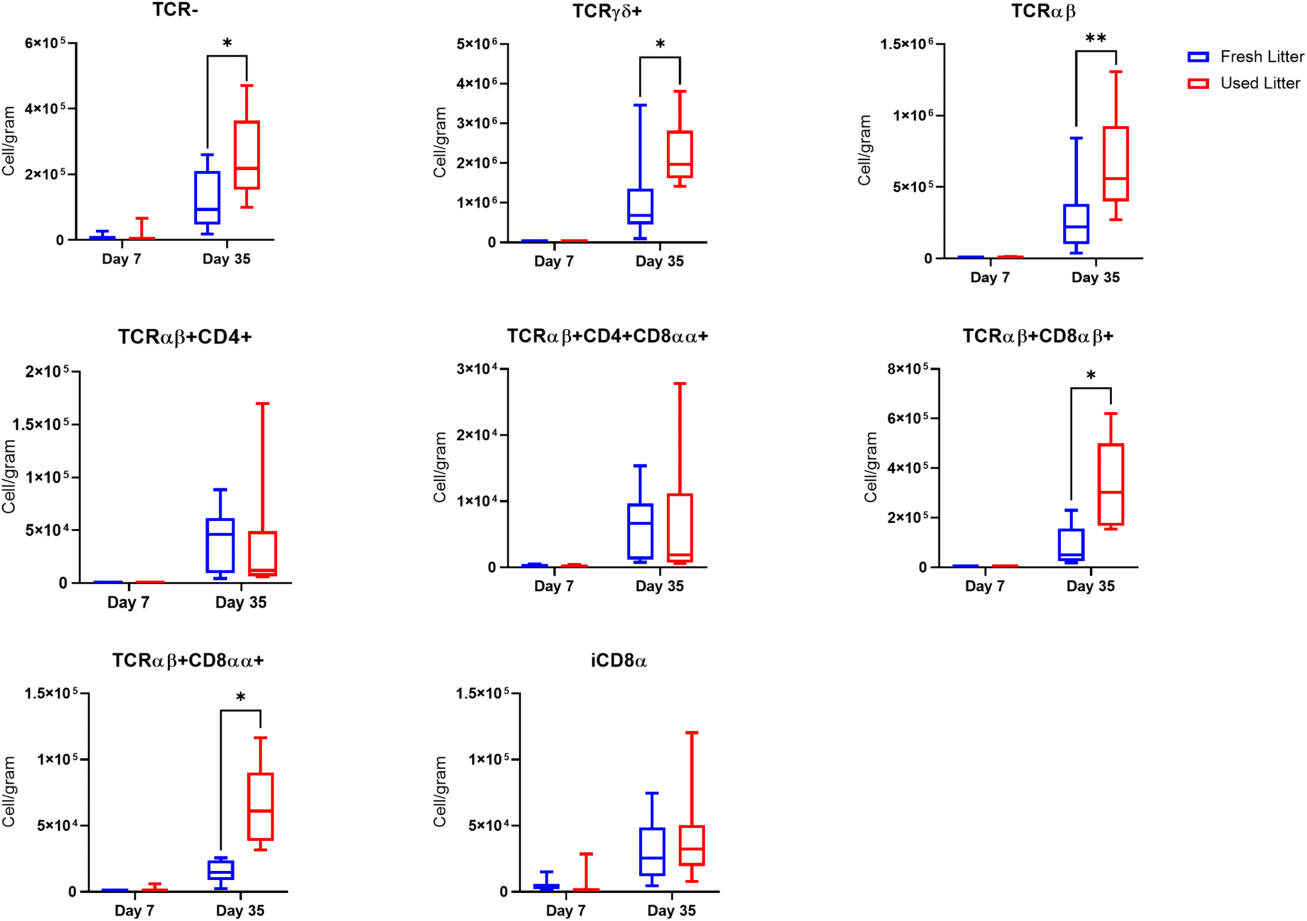
Effect of litter type on ileal intraepithelial lymphocyte (IEL) populations at day 7 and day 35. The box plot shows the distribution of the data, and the central line shows the median (n = 8), while whiskers depict variability outside the upper and lower quartiles. Statistical significance was determined using the Mann-Whitney U test. * p < 0.05, ** p < 0.01.

**Fig 7:**
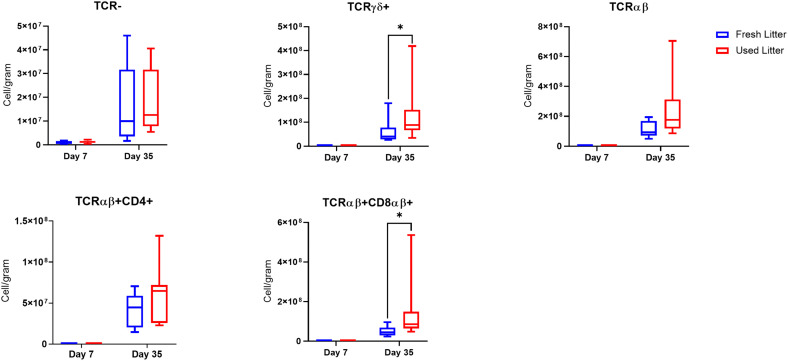
Effect of litter type on splenic immune cell populations at day 7 and day 35. The box plot shows the distribution of the data, and the central line shows the median (n = 8), while whiskers depict variability outside the upper and lower quartiles. Statistical significance was determined using the Mann-Whitney U test. * p < 0.05.

At day 7, no significant differences were observed in the number of ileal IEL subsets between layer chicks reared on fresh and used litter (Fig 6). However, by day 35, several IEL populations were significantly increased in layer chicks reared on used litter, including TCR ⁻ , TCRγδ ⁺ , TCRαβ ⁺ , TCRαβ ⁺ CD8αβ ⁺ , and TCRαβ ⁺ CD8αα⁺ cells (p < 0.05) (Fig 7).

Similar trend was observed in the spleen, where the number of immune cells was comparable at day 7 (Fig 7). Then by day 35, layer chicks raised on used litter harbored increased numbers of TCRγδ⁺ and TCRαβ ⁺ CD8αβ⁺ cells (*p* < 0.05).

## Discussion

The present study demonstrates that the reused litter significantly shaped early intestinal microbial community structure and the immune cell populations in layer chicks. Layer chicks reared on fresh litter exhibited greater body weights during the grower phase, whereas used litter exposure was associated with distinct microbial profiles at day 7 and enhanced expansion of CD8-cytotoxic and γδ T-cell populations by day 35. Together, these results suggest that early environmental microbial exposure through litter may influence the progression of gut microbial colonization and subsequent immune system development.

The higher body weight observed in layer chiks reared on fresh litter in present study may be attributed to differences in litter quality, microbial load, and environmental conditions. Fresh litter provides a cleaner and more hygienic rearing environment, reducing exposure to harmful microorganisms, ammonia, and other contaminants that may impair growth performance. In contrast, reused litter may accumulate moisture, pathogens, and noxious gases over time, which can negatively affect feed intake, respiratory health, and nutrient utilization. As a result, layer chicks reared on new litter are likely to experience less physiological stress and better growth conditions, leading to improved body weight gain. These findings aligns with reports suggesting that cleaner litter conditions may support improved growth performance through reduced environmental microbial load and lower subclinical immune stimulation in broilers [17]. However, findings across studies are inconsistent. Ekunseitan et al. [18] and Hussain et al. [19] reported greater weight gain in broiler chicken reared on recycled litter, suggesting that early microbial exposure may, under certain conditions, enhance gut adaptation and nutrient utilization. In contrast, Abougabal [20] found no statistically significant differences in weight gain between litter types, although numerically higher weights were observed in broilers on new litter. Collectively, these contrasting reports indicate that the effects of litter reuse on body weights are context-dependent and influenced by factors such as chicks age, litter management, housing conditions, and microbial load. Under the conditions of the present study, fresh litter provided a growth advantage during the starter phase.

Following the observed differences in growth performance, our cecal microbiota analysis indicates that litter type primarily influenced early microbial colonization rather than long-term community structure. At day 7, significant differences in both alpha and beta diversity between fresh and used litter groups demonstrate that litter background strongly shaped initial community assembly. This early divergence reflects the microbial seeding effect of used litter, which introduces an established anaerobic community into the premature gut. Similar findings have been reported by Cressman et al. [2] and De et al. [21] who showed that reused litter accelerates microbial succession and promotes earlier stabilization of anaerobic populations, while Ekunseitan et al. [18] also documented litter-driven differences in early microbial richness. However, by day 35, no significant differences were detected, indicating convergence of microbial communities over time. This pattern is consistent with reports that host age becomes a dominant determinant of gut microbial structure during maturation [6,21].

In the present study, the cecal microbiota of layer chicks was dominated at all sampling points by Bacillota (Firmicutes), with secondary contributions from Bacteroidota (Bacteroidetes), Actinomycetota (Actinobacteria), Pseudomonadota (Proteobacteria), and Cyanobacteriota, a pattern that broadly agrees with previous reports in both layers and broilers [22–24]. At day 7, both fresh and used litter groups showed Bacillota as the most abundant phylum, followed by Pseudomonadota, which is consistent with the notion that early‑life cecal communities in chickens are still maturing and often characterized by relatively higher proportions of Proteobacteria and lower overall diversity compared with adult broilers [25]. By day 35, the microbiota in both litter systems shifted toward a more mature community structure [26]. In this stage, Bacillota and Bacteroidota became the two dominant phyla, followed by Actinomycetota, while Proteobacteria or Cyanobacteriota occurred at relatively lower abundances. This configuration closely mirrors phylum-level profiles reported in adult laying hens and market-age broilers, where Bacillota and Bacteroidetes typically constitute the majority of cecal bacterial sequences [27]. Importantly, comparing fresh and used litter within each age indicates that reusing litter did not alter the core phylum‑level architecture of the cecal microbiota, Bacillota remained dominant in all groups and Bacteroidota emerged as the second ranked phylum by 35 days irrespective of litter history [2,6]. Instead, litter treatment influenced the relative ranking of minor phyla, with used litter layer chicks showing a higher prominence of Actinomycetota at day 7 and Cyanobacteriota at day 35, while fresh litter layer chicks tend to have Pseudomonadota more prominent at both ages. These results indicate that used litter does not markedly disrupt the typical development of the cecal microbiota dominated by Bacillota and Bacteroidota, but rather produces minor shifts in less abundant phyla without altering the characteristic microbiome maturation observed in chickens.

The LEfSe analysis at day 7 further revealed that layer chicks reared on used litter were enriched with Firmicutes-associated anaerobic taxa, including Oscillospirales, Ruminococcaceae, Faecalibacterium, and Clostridia-related groups. These taxa are widely recognized as predominant members of the cecal microbiota and are associated with mature, fermentative communities [23,28]. Consistent with our findings, Wang et al. [6] reported greater abundance of Faecalibacterium and Oscillibacter in reused litter systems, while Cressman et al. [2] observed Clostridiales dominance under used litter conditions. In contrast, fresh litter layer chicks showed enrichment of facultative and early colonizing taxa such as Enterococcus, Proteus, Morganellaceae, Bifidobacteriaceae, and Actinobacteria, which are commonly associated with early-stage microbial establishment [28]. These findings suggest that used litter accelerates early microbial maturation, whereas fresh litter supports a more transitional early-life microbial profile, although these differences diminish as birds mature.

The intestinal microbiota plays a crucial role in maintaining gut health by facilitating the digestion of dietary components, inhibiting pathogen colonization through nutrient competition and the production of bacteriocins and antimicrobial compounds, and modulating the development and function of the host immune system [29]. In the present study, the cecal of layer chicks raised on the used litter harbor highly diverse unique microbial profiles at day 7 that was reflected on the immune cell populations in the intestine and spleen. By day 35 of age, the ileal of used litter birds harbor induced numbers of TCRαβ ⁺ CD8αα ⁺ , TCRαβ ⁺ CD8αβ ⁺ , TCRγδ ⁺ , and TCR ⁻ IELs. In mice, the microbiota are critical for the development and maintenance of the IEL population [30,31]. For instance, Microbial signals activate NOD2 in dendritic and epithelial cells, leading to IL-15 production which induce survival and homeostasis of TCRαβ ⁺ CD8αα⁺ cells [29]. Consistent with this pattern, splenic CD8αβ⁺ and γδ T cells were also elevated at day 35 in used litter layer chicks, indicating that early intestinal immune priming may extend to systemic immune organs with age. These findings are consistent with the work of Lee et al. [32] demonstrated that commercial broilers raised on used litter exhibited enhanced immune activation, including higher serum nitric oxide levels, stronger spleen cell proliferative responses, and alterations in IEL subpopulations compared with broilers reared on Fresh litter. These observations support the interpretation that used litter promotes progressive mucosal immune maturation, through sustained microbial antigen exposure.

To our knowledge, this study represents the first controlled evaluation of the effects of fresh and reused litter on both cecal microbiota composition and immune-cell populations in layer chicks. The findings provide novel evidence that litter environment is associated with early microbial colonization patterns and immune-cell development, thereby expanding current knowledge beyond the broiler-focused literature. Although baseline litter microbiota was not characterized and replicated room-level units were not included, birds were housed in separate rooms to reduce cross-contamination. Future studies with baseline litter profiling, replicated units, and challenge models will help confirm the long-term biological significance of this study.

## Conclusion

Reused litter influenced early cecal microbial colonization and was associated with enhanced expansion of ileal intraepithelial lymphocyte subsets, including TCR ⁻ , TCRγδ ⁺ , TCRαβ ⁺ , TCRαβ ⁺ CD8αβ ⁺ , and TCRαβ ⁺ CD8αα ⁺ , as well as increased splenic TCRγδ⁺ and TCRαβ ⁺ CD8αβ⁺ populations during subsequent immune maturation in layer chicks. Although treatment-associated microbial differences were noticeable in early rearing phase, cecal microbiota profiles converged by day 35, with Bacillota remaining dominant and Bacteroidota increasing with age. Future studies should assess whether these microbial and immune changes influence laying performance and enhance resistance to enteric pathogens under controlled challenge conditions.
